# An evidence-based tailored eHealth patient education tool for patients with knee osteoarthritis: protocol for a randomized controlled trial

**DOI:** 10.1186/s12891-022-05212-0

**Published:** 2022-03-22

**Authors:** Kangping Song, Siyi Zhu, Xiaona Xiang, Lin Wang, Suhang Xie, Huizhen Liu, Wenjie Yang, Chengqi He

**Affiliations:** 1 Rehabilitation Medicine Center, Department of Rehabilitation Medicine, West China Hospital, Sichuan University, Chengdu, 610041 Sichuan PR China; 2 School of Rehabilitation Sciences, West China School of Medicine, Sichuan University, Chengdu, China; 3 Rehabilitation Key Laboratory of Sichuan Province, West China Hospital, Sichuan University, Chengdu, China; 4 Centre for Biostatistics, Design, Measurement and Evaluation (CBDME), West China Hospital, Sichuan University, Chengdu, China

**Keywords:** Osteoarthritis, Patient education, Telemedicine, Patient medication knowledge, Patient satisfaction

## Abstract

**Background:**

Osteoarthritis is a common and disabling condition that places heavy burden to individuals and healthcare systems. Patient education is a facilitator in the treatment decision making process, aiming to develop a treatment plan for the disease management. Electronic health (eHealth) is an alternative forum for the delivery of patient education and given the prevailing of eHealth in healthcare, introducing patient education programs using the technology has the potential to improve patient engagement, self-management and outcomes in patients with osteoarthritis. The study will evaluate the efficacy of eHealth patient education tool on patients’ perception of knee osteoarthritis and treatment options, satisfaction and compliance to treatments.

**Methods:**

This study is a prospective randomized controlled trial with a 1:1 allocation in two groups. We will recruit 216 patients diagnosed with knee osteoarthritis from the outpatient physiatry/physiotherapy clinic at West China Hospital, Sichuan University in Southwest China. Both groups will receive usual care and additionally, the intervention group will use eHealth patient education tool during the process. Measurements will be taken at baseline, post-intervention, 1 month, 3- and 6-months follow-up. Primary outcome will be patients’ knowledge about disease and treatment options, measured by the validated osteoarthritis patient knowledge questionnaire. Secondary outcomes include patients’ satisfaction with the consultation, the eHealth patient education tool, and their trust of the physiotherapist.

**Discussion:**

The eHealth patient education tool is designed to provide participants with an innovative model of care delivery and this trial will assess the efficacy of the tool and whether this new model of patient education will have the potential to increase patient knowledge and empower self-management. Results collected from this study will further inform future research employing eHealth tool as interventions for the management of a range of other chronic conditions and help participants in communities or rural areas having the equal access to health care services.

**Trial registration:**

This study was prospectively registered on the Chinese Clinical Trials Registry (ChiCTR2100051083) registered 12.09.2021.

**Supplementary Information:**

The online version contains supplementary material available at 10.1186/s12891-022-05212-0.

## Background

Osteoarthritis is a common and disabling condition that has an increasing health burden, with notable implications for people older than 60 years and healthcare systems [1]. Global percentage change in years lived with disability between 2006 and 2016 was 31.5% [2]. In China, around 61.2 million individuals had osteoarthritis recorded in 2017 [3, 4]. Treatment options range from non-pharmacological methods, such as education and self-management, physical activity and exercise, weight loss and physical modalities, to invasive treatments such as intra-articular injections and joint replacement surgery [1]. However, patients may be exposed to different benefits and risks receiving these treatments; therefore, the knowledge on osteoarthritis, how to manage, when and how to make decisions, should be incorporated into a discussion in the development of a treatment plan between physicians and patients.

Patient education that meets patients’ needs have been proved effective in improving patients’ performance in outcomes, knowledge on a disease, and facilitation of decision making [5]. Patient education was strongly recommended by 12 of 15 evidence-based practice guidelines included in a systematic review [6]. Distinct from providing health information alone, evidence-based patient education, a systematic approach combining current medical best evidence, are provided for patients to help understand a disease and its progressive nature, and the management alternatives and protection strategies to facilitate self-care [7, 8]. A recent published trial reported [9] that the knowledge uptake was significantly improved in patients receiving a poster summarizing a clinical practice guideline on conservative treatment options for knee osteoarthritis compared to that in usual care group. A more recent review [10] linked patient education to improved pain and function outcomes compared with usual care in patients with knee osteoarthritis. Despite the documented benefits of patient education, adoption among physicians and compliance with the recommendations of the guideline varies remarkably across different healthcare settings. Studies of patient education have significant heterogeneity in assessing the potential impact of these education programs on health and well-being of participants with osteoarthritis, from which existed inconsistent effectiveness of patient education [11]. Besides, there is always a lack of support in the delivery of patient education, which aims to improve outcomes for patients with osteoarthritis [11]. Two studies recommended that patient education with incorporation of recommendations in clinical practice guidelines and decision-making support has the potential to minimize variation in delivery of care [12, 13]. The development and application of such education tools has huge values in improving patient comprehension and compliance with current practice guidelines.

To elicit maximum impact, electronic health (eHealth), defined as “the use of information and communication technologies for health” by WHO [14], is an alternative forum for the delivery of patient education. Given the prevalence of eHealth in healthcare, introducing patient education programs by using the technology provides tremendous flexibility in disseminating health information, where participants have the choice to access information at any given time without the limitation of location [15]. The positive role of eHealth acting at improving some self-management and adherence to treatment in patients with various diseases has been well recorded [16]. Accumulating evidence indicated that self-management and exercise programs for knee osteoarthritis powered by eHealth approaches significantly improved pain, physical function and quality of life compared to usual care [8, 17]. Similar effects are expected in the patient education of knee osteoarthritis. But for some participants using the eHealth intervention in an undesired way, the diminished effect of these interventions was reported, into which gaining an insight should become one of main focuses in any research involved eHealth interventions [18]. Therefore, designing an eHealth intervention tool incorporating of advanced feedback mechanism like regular reminders and active strategies is necessary. However, a study investigating the effect of a patient education program incorporating the recommendations of clinical guidelines (e.g., OARSI guidelines for the non-surgical management of knee osteoarthritis) with decision-making support through an eHealth approach (developed with participants actively involved) on patients’ perception of knee osteoarthritis as a disease and treatment options, satisfaction and compliance to treatments has not been performed in a population with knee osteoarthritis, specifically for whom live in Southwest China (with the highest prevalence rate, especially in rural areas and communities).

## Methods

### Aim

The primary objective of this study is to evaluate the impact of a physiotherapist-delivered eHealth patient education tool incorporating the recommendations of clinical guidelines (OARSI and COA guidelines) [19, 20] on patient knowledge, to what extent patients with knee osteoarthritis understand the condition and the efficacy of non-pharmacological treatment options.

The secondary objective is to assess patient satisfaction during the process of using the eHealth patient education tool in patients with knee osteoarthritis.

### Study design

This study will be a prospective randomized controlled trial (RCT) comparing an eHealth patient education tool to usual care for knee osteoarthritis. This protocol has been designed according to the Standard Protocol Items: Recommendations for Interventional Trials (SPIRIT) statement [21]. A completed SPIRIT checklist can be found in Additional file 1. In addition, we have used Tidier checklist [22] to help report our intervention section and the checklist could be found in Additional file 5.

### Participants and setting

We will recruit 216 participants referred to the outpatient physiatry/physiotherapy clinic at West China Hospital, Sichuan University in Southwest China. Ethics approval was obtained from Ethics Committee on Biomedical Research, West China Hospital of Sichuan University, and prospectively registered on the Chinese Clinical Trials Registry (ChiCTR2100051083) registered 12.09.2021. Potential participants will be identified from clinic schedules and asked on site to assess their eligibility and to obtain written consent.

To be included, potential participants must present with one of the following: (1) be aged over 45 years, (2) have knee osteoarthritis (Kellgren-Lawrence grade 2 or greater) with symptoms lasting for at least 3 months, (3) is able to provide informed consent, (4) be fluent in verbal and written Chinese, (5) with adequate hearing and eyesight, (6) own an internet-capable device and with access to internet, (7) is not included in the waitlist of knee replacement surgery, (8) be in an independent ambulatory status.

Potential participants will be excluded if they have any of the following: (1) not able to read or speak Chinese, (2) had a history of clinic visits or an appointment with any physician/therapist/healthcare professional for evaluation of knee osteoarthritis, (3) had a diagnosis of fibromyalgia or a systematic arthritic condition, (4) had a history of surgery related to knee, hip and other joints.

### Randomization and allocation

Eligible participants will be randomized with a one-to-one intervention allocation to the intervention (*n* = 108) or usual care (*n* = 108) group at the time of consent. Allocation concealment will be blinded and performed in the order of recruitment using a computer-generated random allocation schedule operated at the Centre for Biostatistics, Design, Measurement and Evaluation (CBDME) of West China Hospital, Sichuan University by a senior statistician after consent has been obtained. Random permuted blocks of sizes 4 or 6 will be employed to ensure participants are allocated to each group equally. Participants, research coordinators, and the eHealth physiotherapist will be notified, via a sealed opaque envelope, on which group the participant has been randomly assigned.

### Blinding

Due to the characteristic of the research, participants and physiotherapists will not be blinded to group allocation and will be aware of the alternative treatment options. The study hypotheses will be blinded to participants. The researcher conducting the data collection and analysis, however, will be blinded to treatment allocation.

### Participant timeline

**Fig. 1:**
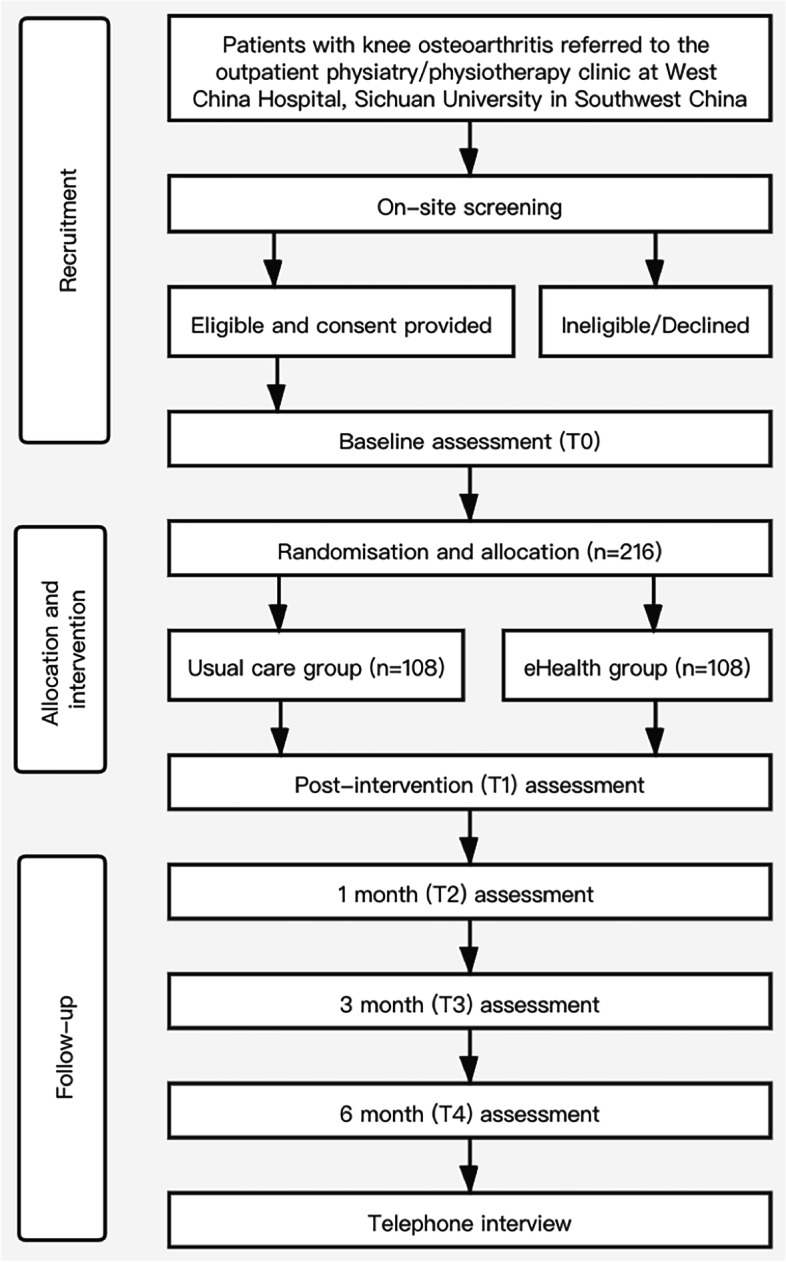
Flow-chart of the study

Table 1 shows the assessments at each time point following the SPIRIT statement [21]. Figure 1 demonstrates the flow chart of the study.

**Table 1:**
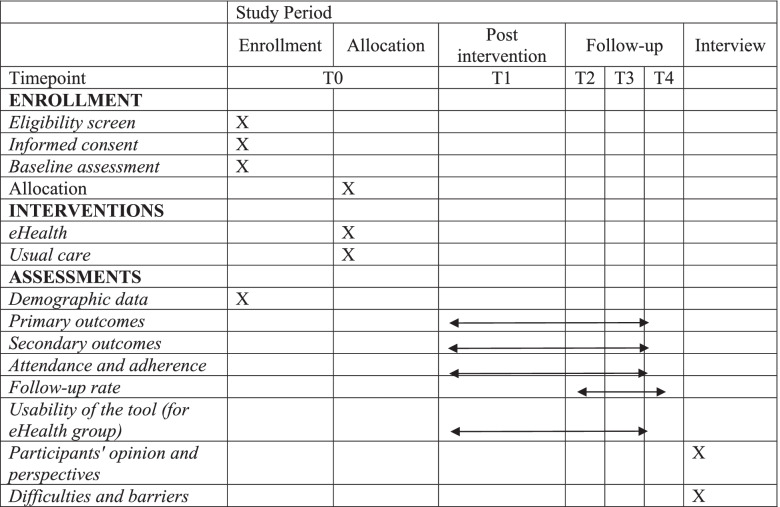
Study assessments at specific time points

### Usual care group

The control group will receive usual care including the wide range of care practices provided in the outpatient clinic at West China Hospital which are unrestricted by study protocols, which is in contrast to standard care may not reflect real-world care [23]. Participants in this group will be required to record treatment received using a log sheet weekly over the course of the study period. The common practice consists of a visit to a physiatrist or physiotherapist at outpatient clinic, verbal information about knee osteoarthritis and treatment options, prescription of pain medication, may involve physical or occupational therapy (e.g., manual therapy, physical agents) and advice about home exercises. All participants in the control group will be contacted via telephone or WeChat messenger (Tencent Holdings Ltd., Shenzhen, China) by an assistant who will provide instructions for the participant on how to finish the follow-up questionnaires and access the planned care they were seeking for knee osteoarthritis.

### eHealth intervention group

Participants assigned to the intervention group will, in addition to usual care, receive an invitation QR code via WeChat messenger to access the eHealth patient education tool built within the WeChat mini programs platform. Participants register themselves using a username and password. The intervention includes instructions on how to efficiently use the tool, information detailing the knowledge of knee osteoarthritis and the recommendations of clinical guidelines including level of evidence supporting each recommendation [24], and advice on healthy lifestyle, in a scheduled video conference with a physiotherapist using Tencent Meeting app (Tencent Holdings Ltd., Shenzhen, China) on a smartphone, a tablet or computer. Participants will be provided with a welcome brochure with an overview of the intervention components, and instructions on how to register and use the eHealth patient education tool and Tencent Meeting app (Web browser, Android or iOS app) at home. The intervention will be delivered within 7 days after the randomization, during which the video consultation with a physiotherapist will last up to 90 min according to the patient’s preference. Automatically generated push notifications within WeChat messenger will be sent to motivate participants to review the information and use the tool twice a week after the consultation for at least 6 months. Additionally, participants have the possibility to get help from the coordinators or via the study hotline regarding any technical problems when using the tool.

An iterative method of collaboration-based persuasive design between healthcare providers and patients is employed to the development of the eHealth patient education tool [18]. In brief, a focus group (*n* = 6, involved 3 patients with knee osteoarthritis, 2 physiotherapists and 1 physiatrist) was initially conducted in two phases during the development process, in which healthcare providers met at Phase I to provide input on the eHealth prototype regarding design and content and patients were invited to Phase II to discuss with healthcare providers regarding literacy demands and usability. Then a pilot-testing of the developed tool will be done among healthcare providers and patients (with a total of 20 participants) in a similar way to the focus group, and the testing version could be found in Additional file 2. The tool includes the following features:

- **Education Center:** information on knee osteoarthritis and treatment options (details could be found in Additional file 2), based on the recommendations of clinical guidelines; exercise advice and videos; physical activity and well-being advice.
- **Personal Information:** the option to record demographic data and history of knee osteoarthritis.
- **Self-Management:** self-monitor symptoms during the study; log the planned care received for knee osteoarthritis and other comorbidities.
- **Schedule and Plan:** set reminders and develop a calendar for the scheduled consultation, and assessments.
- **Communication and Help Center:** contact with an assigned physiotherapist or healthcare professional; an overview of the educational tool.

Participants who cannot attend the consultation will be excluded after contact has been attempted three times. One day after the consultation with the physiotherapist, all participants will receive the follow-up questionnaires on their WeChat. A reminder will be sent if participants are found non-responding after 1 week. Intervention fidelity will be assessed to rate the degree of involvement of a patient within 24 h after the physiotherapist-delivered consultation. As a participant reads information or watches a video, analytics built into the server website will collect data on what type of information and how long the participant spent on that. The chief research coordinator, program developer, and research assistance conducting data collection have access to the website and are able to view the analytics for each participant.

### Outcome assessment

Outcome measures will be assessed at five time points through an online link powered by Wenjuanxing (www.wjx.cn, a website like Surveymonkey), namely at baseline (T0), post-intervention/consultation (T1), 1 month (T2), 3- and 6-months (T3 and T4) follow-up after the randomization.

#### Primary outcome

Primary outcomes included knowledge about disease and treatment options. The validated osteoarthritis (OA) patient knowledge questionnaire (PKQ-OA) [25] will be used, modified, and adapted to reflect the key learner content covered in the eHealth patient education tool. The questionnaire comprises 7 single choice and 14 multiple choice questions with 31 correct answers and details of the questionnaire could be found in Additional file 4. We modified the PKQ-OA closely according to the patient education information provided in the education center and the questions are relevant with the evidence-based information. Total knowledge scores will be obtained by summing the scores, range from 0 to 21. Five patients with knee osteoarthritis and three healthcare professionals will provide input on the prototype of eHealth tool regarding design, content and usability, from which data collected will be used to finalize the tool for the trial. Higher scores indicate greater disease knowledge, and scoring above 50% on the knowledge portion of the questionnaire will be classified as adequately informed [26].

#### Secondary outcome

The secondary outcome measures will track patient satisfaction with the consultation, the eHealth patient education tool, and their trust of the physiotherapist. Maintaining a similar format as the adopted survey [27], the questionnaire comprises 6 closed questions and 2 open questions for participants in both groups and details of the questionnaire could be found in Additional file 4.

#### Other measures

Participants’ demographic data, history of diseases and health behavior information will also be collected. Potential “inadequate health literacy level” in participants will be screened using 3 questions: “how confident are you filling out questionnaires by yourself?” (“Confident with Questionnaires”), “Do you need someone to help you read? If yes, how often?” (“Read Assistance”) and “Do you have obstacles learning about your medical condition due to your inability to read? If yes, how often?” (“Problem Reading”) [28, 29]. In the process evaluation, measures of recruitment rate, attendance and follow-up rate will be monitored and recorded. Reasons for exclusions, declining participation and the drop-out of participants will be noted through the trial.

Participants in the eHealth intervention group will receive additional questions on the usability of the tool, including 13 closed questions and 3 opened-ended questions [30] and details of the questionnaire could be found in Additional file 4. Web-based analytics generated by the educational tool will be used to assess the utilization of the tool, which include data on content with which they engage, length of time spent on different content, and satisfaction ratings or feedback.

The measure of opinion and perspectives for participating the trial will be assessed via a phone- recorded interview (semi-structured) in both the eHealth and usual care group with the interviewee’s permission, for which a guide with open questions will be used to provide structure. The phone call will be made by a researcher who have experiences in qualitative interviews and is independent of the conduction of this trial. Two researchers (independent of the conduction of this trial) will be instructed by the interviewer to independently read and code transcribed texts line-by-line, from which codes generated will be sorted into themes and discussed to gain an overall understanding of any issue on difficulties and barriers to complete the trial. Any discrepancy will be resolved by the interviewer (senior researcher). During the trial, adverse events, medications and any other care received will be recorded using a log sheet, a sample could be found in Additional file 3.

##### Data integrity and monitoring

To assess trial safety and ensure that the best interests of participants will be observed at all times, an independent Data Safety Monitoring Committee will be assembled. Once 10, 50, and 80% of the sample size is reached, a data quality audit will be performed during the trial being conducted. Further, data will be stored in encrypted spreadsheets on secured servers hosted by the West China Hospital of Sichuan University, in which any potential risk of omissions and errors will be regularly scrutinized, and then exported to a statistical software for analysis by a statistician blinded to group allocation. All data collected in this trial will be restricted to the principal investigator and specific members of the research team using the backend of the database or servers.

##### Sample size

Sample size calculation was conducted based on the between-group difference on primary outcome, assuming that participants in the intervention group will have a correct response rate of 55.3% compared to that of 39.5% in the control group [27]. Based on a power of 95% and a 5% significance level (two-sided test), a value of 0.5 SD was used to ensure a large enough sample size. A Z-score of 1.96 was determined by applying these parameters. To allow for a dropout rate of 20%, a sample size of 108 per group (total of 216 participants) is required. Calculations were performed with PASS 15.0 (NCSS, LLC).

### Statistical analysis

Summary statistics will be calculated and reported in accordance with the Consolidated Standards of Reporting Trial (CONSORT). The baseline comparability between groups will be tested among descriptive characteristics, as well as baseline outcome measures. The intention to treat analysis will be performed by a blinded statistician. Missing values will not be imputed unless the amount of that for an outcome is over 5%, for which multiple imputation will be performed. Quantitative data will be expressed as the Mean (SD), and number plus percentage will be used to describe nominal data. The treatment effect will be evaluated by the change in primary outcome between group analyses, using independent t-test and ANCOVA for quantitative variables and the χ^2^ test or the Fisher exact test to adjust for demographic data such as age, sex, education background, literacy level, joint symptoms, comorbidities, type and duration of treatment on change in outcomes. Repeated measure effect will be evaluated by the repeated measures ANOVA or generalized estimating equation, where applicable. Correlations between data on usability of the educational tool and patient reported outcomes will be analyzed using linear regression analysis within the intervention group. Constructivist grounded theory and a relational ethics lens will guide the plan for qualitative analysis [31]. The QSR NVivo 12 software will be used to organize and store the qualitative data. Memo-writing will be used to develop and refine key conceptual categories, outline themes under which each category lies and analyze relationships across these key conceptual categories. This conceptual framework will draw attention to understanding user experiences and engagement with the intervention in a context of relational settings. A statistical analysis plan will be posted on a public data repository before analysis.

### Ethical considerations and dissemination

The protocol with any modifications before implementation will be re-submitted to the Human Research Ethics Committee of West China Hospital, Sichuan University, and amendments to the protocol will be updated in the trial registries and outlined at the section of dissemination. The confidentiality and privacy of data retrieved from this trial will be protected in accordance with clinical research regulations developed by National Health Commission and the International Council for Harmonisation of Technical Requirements for Pharmaceuticals for Human Use (ICH) [32, 33]. The final report or any presentations of this trial will be presented as aggregated results in which individual participants will not be identifiable.

A short report summarizing associated results will be presented to participants involved. Results of this trial will be presented at conferences and published in forms of peer-reviewed journal manuscripts. All researchers in this trial will be considered as co-authors of future publications according to their contribution. The protocol of this trial will be posted on the website of the clinical trials registrations and Human Research Ethics Committee.

## Discussion

We present a protocol for a randomised clinical trial involving an eHealth patient education tool for participants with knee osteoarthritis compared to usual care. The tool is designed to provide participants with an innovative model of care delivery without having them come to hospital and reducing health burden caused by knee osteoarthritis. It is estimated that approximately 1.71 billion people around the globe have musculoskeletal conditions, among which osteoarthritis accounted for 343 million costing billions of dollars to economies annually [34]. In China, the total number of YLDs for knee osteoarthritis reached to 4,149,628, and YLDs rate was per 968 per 100,000 population, in which Southwest China had the highest YLD rate from knee osteoarthritis accounted for 1653 per 100,000 population [35].

If the eHealth educational tool is found to be effective, this new model of patient education has.

the potential to increase patient knowledge and empower self-management by achieving better health outcomes, informing decision-making, and promoting efficiency of communications [36]. Results collected from this study will further inform future research employing eHealth tool as an intervention for the management of a range of other chronic conditions, and support resource efficiency to ensure that participants in communities or rural area have the equal access to health care services.

A potential limitation of this trial is some participants aged over 60 may have barriers (e.g., technological skill, attitudes towards eHealth) to proficiently use the eHealth patient education tool. Furthermore, a potential mode effect due to different models of device used by participating this study is another limitation and potential source of bias. Therefore, improvements to tackle these barriers and increase acceptance of technology in this population are currently underway.

## Supplementary Information

**Additional file 1.**

**Additional file 2.**

**Additional file 3.**

**Additional file 4.**

**Additional file 5.**

## Data Availability

Not applicable.

## Abbreviations

eHealth: Electronic health
WHO: World Health Organization
OARSI: Osteoarthritis Research Society International
COA: Chinese Orthopaedic Association
RCT: Randomized controlled trial
SPIRIT: Standard Protocol Items: Recommendations for Interventional Trials
CBDME: Centre for Biostatistics, Design, Measurement and Evaluation
QR: Quick response
OA: Osteoarthritis
PKQ-OA: Osteoarthritis patient knowledge questionnaire
SD: Standard deviation
CONSORT: Consolidated Standards of Reporting Trial
ANCOVA: Analysis of Covariance
ANOVA: Analysis of Variance
ICH: International Council for Harmonisation of Technical Requirements for Pharmaceuticals for Human Use
YLDs: Years Lived with Disability

## References

[1] Hunter DJ, Bierma-Zeinstra S (2019). Osteoarthritis. Lancet.

[2] Vos T, Abajobir AA, Abate KH, Abbafati C, Abbas KM, Abd-Allah F (2017). Global, regional, and national incidence, prevalence, and years lived with disability for 328 diseases and injuries for 195 countries, 1990–2016: a systematic analysis for the global burden of disease study 2016. Lancet.

[3] Long H, Zeng X, Liu Q, Wang H, Vos T, Hou Y (2020). Burden of osteoarthritis in China, 1990–2017: findings from the global burden of disease study 2017. Lancet Rheumatol.

[4] Safiri S, Kolahi AA, Smith E, Hill C, Bettampadi D, Mansournia MA (2020). Global, regional and national burden of osteoarthritis 1990-2017: a systematic analysis of the global burden of disease study 2017. Ann Rheum Dis.

[5] Lopez-Olivo MA, Ingleshwar A, Volk RJ, Jibaja-Weiss M, Barbo A, Saag K (2018). Development and pilot testing of multimedia patient education tools for patients with knee osteoarthritis, osteoporosis, and rheumatoid arthritis. Arthritis Care Res.

[6] Nelson AE, Allen KD, Golightly YM, Goode AP, Jordan JM (2014). A systematic review of recommendations and guidelines for the management of osteoarthritis: the chronic osteoarthritis management initiative of the U.S. bone and joint initiative. Semin Arthritis Rheum.

[7] Bunge M, Mühlhauser I, Steckelberg A (2010). What constitutes evidence-based patient information? Overview of discussed criteria. Patient Educ Couns.

[8] Safari R, Jackson J, Sheffield D (2020). Digital self-management interventions for people with osteoarthritis: systematic review with Meta-analysis. J Med Internet Res.

[9] Squiers M, Nelms NJ, Davis AT, Halsey DA, Slauterbeck JR, Blankstein M. A Poster Summarizing the American Academy of Orthopaedic Surgeons Knee Osteoarthritis Clinical Practice Guideline Is a Powerful Tool for Patient Education: A Randomized Controlled Trial. J Arthroplast. 2021;36(1):102-6.e5. 10.1016/j.arth.2020.07.007 32863075

[10] Goff AJ, De Oliveira Silva D, Merolli M, Bell EC, Crossley KM, Barton CJ. Patient education improves pain and function in people with knee osteoarthritis with better effects when combined with exercise therapy: a systematic review. J Phys. 2021;67(3):177-89. 10.1016/j.jphys.2021.06.011 34158270

[11] Kroon FP, van der Burg LR, Buchbinder R, Osborne RH, Johnston RV, Pitt V. Self-management education programmes for osteoarthritis. Cochrane Database Syst Rev. 2014;(1):Cd008963. 10.1002/14651858.CD008963.pub2 PMC11104559 24425500

[12] Carlson VR, Ong AC, Orozco FR, Hernandez VH, Lutz RW, Post ZD (2018). Compliance with the AAOS guidelines for treatment of osteoarthritis of the knee: a survey of the American Association of hip and Knee Surgeons. J Am Acad Orthop Surg.

[13] Meiyappan KP, Cote MP, Bozic KJ, Halawi MJ (2020). Adherence to the American Academy of Orthopaedic surgeons clinical practice guidelines for nonoperative management of knee osteoarthritis. J Arthroplast.

[14] World Health Organization (2021). Digital health.

[15] Slater H, Dear BF, Merolli MA, Li LC, Briggs AM (2016). Use of eHealth technologies to enable the implementation of musculoskeletal models of care: evidence and practice. Best Pract Res Clin Rheumatol.

[16] Marcolino MS, Oliveira JAQ, D'Agostino M, Ribeiro AL, Alkmim MBM, Novillo-Ortiz D (2018). The impact of mHealth interventions: systematic review of systematic reviews. JMIR mHealth and uHealth.

[17] Schäfer AGM, Zalpour C, von Piekartz H, Hall TM, Paelke V (2018). The efficacy of electronic health–supported home exercise interventions for patients with osteoarthritis of the knee: systematic review. J Med Internet Res.

[18] Kelders SM, Kok RN, Ossebaard HC, Van Gemert-Pijnen JE (2012). Persuasive system design does matter: a systematic review of adherence to web-based interventions. J Med Internet Res.

[19] Association CO (2018). Guidelines for the diagnosis and treatment of osteoarthritis (2018 edition). Chin J Orthop.

[20] Bannuru RR, Osani M, Vaysbrot E, Arden N, Bennell K, Bierma-Zeinstra S (2019). OARSI guidelines for the non-surgical management of knee, hip, and polyarticular osteoarthritis. Osteoarthr Cartil.

[21] Chan A-W, Tetzlaff JM, Altman DG, Laupacis A, Gøtzsche PC, Krleža-Jerić K (2013). SPIRIT 2013 statement: defining standard protocol items for clinical trials. Ann Intern Med.

[22] Hoffmann TC, Glasziou PP, Boutron I, Milne R, Perera R, Moher D (2014). Better reporting of interventions: template for intervention description and replication (TIDieR) checklist and guide. BMJ (Clinical research ed).

[23] Thompson BT, Schoenfeld D (2007). Usual care as the control group in clinical trials of nonpharmacologic interventions. Proc Am Thorac Soc.

[24] OARSI. Patient Summary- Non Surgical Treatment of Knee Osteoarthritis. Available from: https://oarsi.org/education/oarsi-resources/patient-summary-non-surgical-treatment-knee-osteoarthritis.

[25] Hill J, Bird H (2007). Patient knowledge and misconceptions of osteoarthritis assessed by a validated self-completed knowledge questionnaire (PKQ-OA). Rheumatology.

[26] Bozic KJ, Belkora J, Chan V, Youm J, Zhou T, Dupaix J (2013). Shared decision making in patients with osteoarthritis of the hip and knee: results of a randomized controlled trial. JBJS.

[27] Squiers M, Nelms NJ, Davis AT, Halsey DA, Slauterbeck JR, Blankstein M (2021). A poster summarizing the American Academy of Orthopaedic Surgeons knee osteoarthritis clinical practice Guideline Is a powerful tool for patient education: a randomized controlled trial. J Arthroplasty.

[28] Chew LD, Griffin JM, Partin MR, Noorbaloochi S, Grill JP, Snyder A (2008). Validation of screening questions for limited health literacy in a large VA outpatient population. J Gen Intern Med.

[29] Sarkar U, Schillinger D, López A, Sudore R (2011). Validation of self-reported health literacy questions among diverse English and Spanish-speaking populations. J Gen Intern Med.

[30] Claassen A, Vliet Vlieland TPM, Busch V, Schers HJ, van den Hoogen FHJ, van den Ende CHM (2019). An electronic health tool to prepare for the first orthopedic consultation: use and usability study. JMIR Form Res.

[31] Charmaz K. Constructing grounded theory. London: Sage; 2014.

[32] Guideline IH (2015). Integrated addendum to ICH E6 (R1): guideline for good clinical practice E6 (R2). Curr Step.

[33] Xiao CM, Li JJ, Kang Y, Zhuang YC (2021). Follow-up of a Wuqinxi exercise at home programme to reduce pain and improve function for knee osteoarthritis in older people: a randomised controlled trial. Age Ageing.

[34] Cieza A, Causey K, Kamenov K, Hanson SW, Chatterji S, Vos T (2020). Global estimates of the need for rehabilitation based on the global burden of disease study 2019: a systematic analysis for the global burden of disease study 2019. Lancet.

[35] Liu Q, Wang S, Lin J, Zhang Y (2018). The burden for knee osteoarthritis among Chinese elderly: estimates from a nationally representative study. Osteoarthr Cartil.

[36] Iribarren SJ, Cato K, Falzon L, Stone PW (2017). What is the economic evidence for mHealth? A systematic review of economic evaluations of mHealth solutions. Plos One.

