# Supplementary material for: An evidence-based tailored eHealth patient education tool for patients with knee osteoarthritis: protocol for a randomized controlled trial
Source: BMC Musculoskelet Disord. 2022 Mar 22;23:274. doi: 10.1186/s12891-022-05212-0 (PMC8939096; doi:10.1186/s12891-022-05212-0)
Supplement: Supplementary file 2 — Additional file 2. [file 12891_2022_5212_MOESM2_ESM.pdf]

Details regarding the information provided to the participants in the education center

Translated version (original in Chinese)

What is knee osteoarthritis?

Osteoarthritis is the most common type of knee arthritis.

The cartilage in the knee joint and the two C-shaped meniscus are the core components to ensure the normal movement, running, jumping and movement of the knee joint.

How knee osteoarthritis happens?

Osteoarthritis occurs over time. People will have the following problems during the aging process:

- The cartilage covering the joint surface becomes thinner.
- The sliding between the articular surfaces is not as smooth as before.
- The joints are more prone to wear.

With aging, osteoarthritis is not inevitable. Osteoarthritis is not simply caused by age and wear. Other factors include simple or repetitive injuries, abnormal activities, metabolic diseases, joint infections, or other joint diseases.

Symptoms?

Symptoms of osteoarthritis usually occur gradually, initially involving only one or a few joints. Finger joints, base of thumb, neck, waist, toes, hips and knees are common areas of involvement.

Pain and morning stiffness are the most common symptoms, usually described as pain deep in the joints, which can be aggravated after climbing or squatting. Some people feel joint stiffness after waking up in the morning or resting, but the stiffness usually subsides within 30 minutes, especially after moving the joints.

Symptoms can remain stable for many years or it can progress rapidly, but most of the time it progresses slowly after symptoms appear. Many people experience some degree of dysfunction.

What causes knee osteoarthritis?

A variety of factors can accelerate the occurrence of knee osteoarthritis. Such as age, heredity, obesity, injury, joint overuse and other joint diseases.

Is it better to get diagnosed early?

Osteoarthritis can remain stable for many years or it can progress rapidly, but most of the time it progresses slowly after symptoms appear. Many people experience some degree of dysfunction. **Therefore, early diagnosis and early treatment are necessary!**

The doctor will diagnose osteoarthritis based on typical symptoms, physical examination, certain blood tests, and X-ray findings of the joints (such as bone hyperplasia and joint cavity stenosis).

Treatment options (Based on the evidence-based medicine guidelines developed by the International Osteoarthritis Association and the Chinese Medical Association)

I. For **all** patients with knee osteoarthritis, the following **primary interventions** or treatments are recommended:

- Therapeutic exercises (resistance training, walking, Tai Chi, etc.)
- Weight loss (it is recommended to reduce 5% of total body weight within 20 weeks)
- Strength exercises of lower limbs
- Water aerobic exercise (swimming, etc.)
- Self-management and education

II. For no other underlying diseases or combined conditions, the following treatment options can be adapted according to the doctor's recommendation:

- Use assistive devices (knee pads, insoles, etc.)
- Use crutches or walkers

- Oral and topical analgesics (nonsteroidal anti-inflammatory medicine, duloxetine, etc.)
- Intra-articular injection of glucocorticoids

III. If the following underlying diseases or combined conditions exist, the following treatment options can be adapted according to the doctor's recommendation:

A. Combined with other underlying diseases (diabetes, hypertension, renal impairment, gastrointestinal diseases, anxiety or obesity, etc.)

- Use assistive devices (knee pads, insoles, etc.)
- Use crutches or walkers
- use topical analgesics (nonsteroidal anti-inflammatory medicine)

B. Polyarticular osteoarthritis combined with underlying diseases (diabetes, hypertension, renal impairment, gastrointestinal disease, anxiety or obesity, etc.)

- Add thermotherapy on the basis of A

Reference *OARSI. Patient Summary- Non Surgical Treatment of Knee Osteoarthritis [Available from: https://oarsi.org/education/oarsi-resources/patient-summary-non-surgical-treatment-knee-osteoarthritis.*

Testing version of eHealth education tool

A. Access to the eHealth patient education tool via invitation QR code

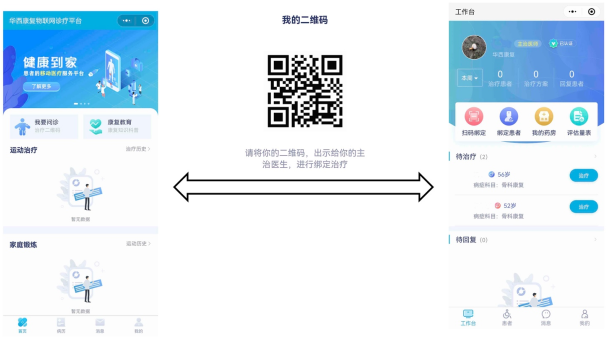

B. Features of the tool

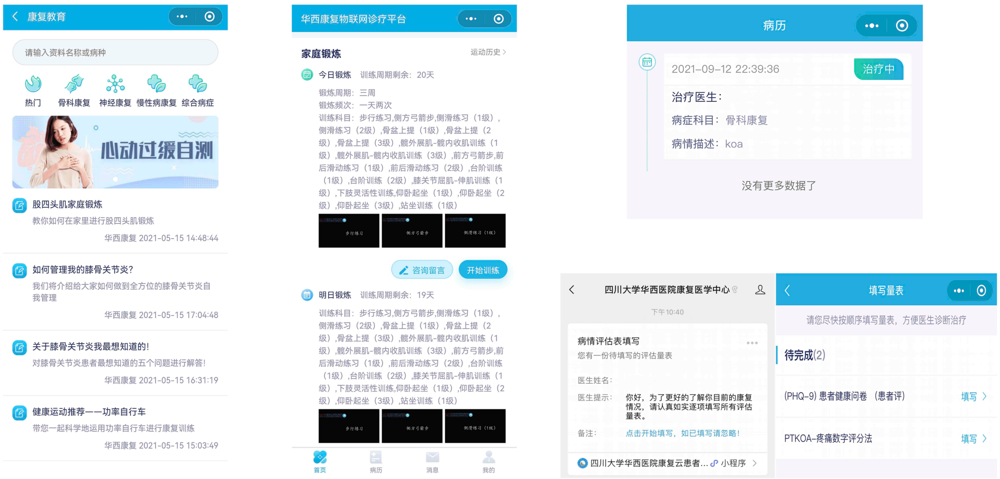

C. Backend of the database

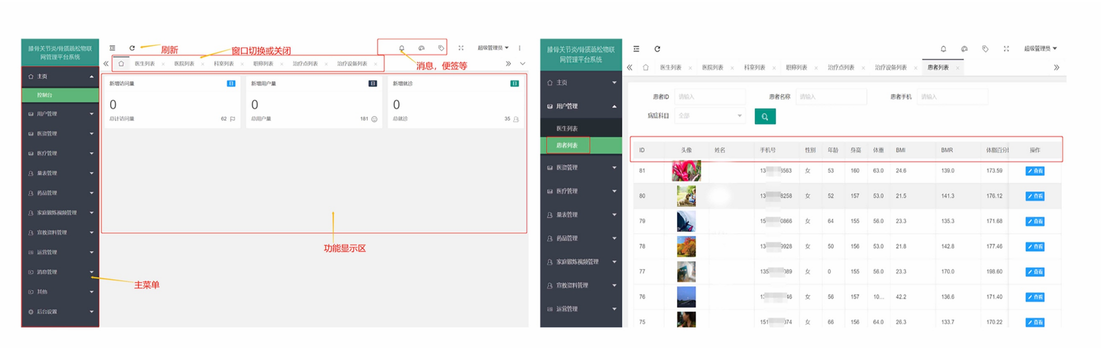

The source of the image: Three images of the eHealth education tool were screenshots from the software, and we have arranged the images according to the actual steps participants and physicians follow.

Permissions to use this image: The images were screenshots from the software, which was designed and developed by our institution and the computer software copyright registration certificate (original document and its English-translated version) is shown below.

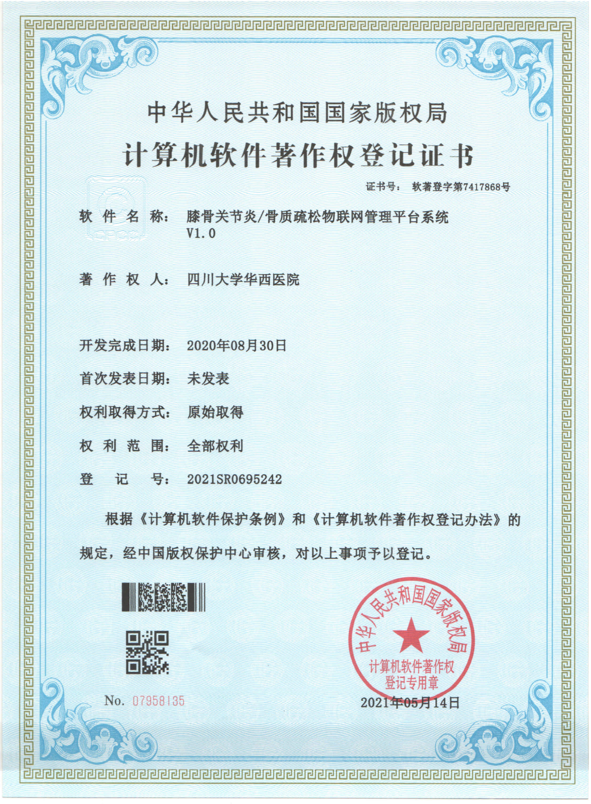

中华人民共和国国家版权局
计算机软件著作权登记证书

证书号： 软著登字第7417868号

软 件 名 称： 膝骨关节炎/骨质疏松物联网管理平台系统 V1.0

著 作 权 人： 四川大学华西医院

开发完成日期： 2020年08月30日

首次发表日期： 未发表

权利取得方式： 原始取得

权 利 范 围： 全部权利

登 记 号： 2021SR0695242

根据《计算机软件保护条例》和《计算机软件著作权登记办法》的规定，经中国版权保护中心审核，对以上事项予以登记。

No. 07958135

中华人民共和国国家版权局 计算机软件著作权 登记专用章

2021年05月14日

National Copyright Administration of the People's Republic of China

Certificate of copyright Registration of computer Software

Certificate number: soft print No. 7417868

Software name: knee osteoarthritis / osteoporosis Internet of things management platform system V1. 0

Copyright owner: West China Hospital of Sichuan University

Development completion date: 30 August 2020

Date of first publication: not published

Mode of acquisition of rights: original acquisition

Scope of rights: all rights

Registration number: 2021SR0695242

In accordance with the regulations on the Protection of computer Software and the measures for the Registration of copyright in computer Software, it is stipulated that the above matters shall be registered after examination and approval by the China copyright Protection Center.

No. 07958135

National Copyright Administration of the People's Republic of China

Computer software copyright Special seal for registration
