# Supplementary material for: An evidence-based tailored eHealth patient education tool for patients with knee osteoarthritis: protocol for a randomized controlled trial
Source: BMC Musculoskelet Disord. 2022 Mar 22;23:274. doi: 10.1186/s12891-022-05212-0 (PMC8939096; doi:10.1186/s12891-022-05212-0)
Supplement: Supplementary file 3 — Additional file 3. [file 12891_2022_5212_MOESM3_ESM.doc]

**Weekly Log Sheet Sample**

**Name: ___________ Group: __________ Date: ___________**

| **Day** | **Planned care/**  **Treatment received** | **Medication**  **(name, dose)** | **Adverse events**  **(pain, soreness, swelling, others)** | **Notes** |
| --- | --- | --- | --- | --- |
| **Monday** |  |  |  |  |
| **Tuesday** |  |  |  |  |
| **Wednesday** |  |  |  |  |
| **Thursday** |  |  |  |  |
| **Friday** |  |  |  |  |
| **Saturday** |  |  |  |  |
| **Sunday** |  |  |  |  |

* Write in each column, in the corresponding day of the week.
