# Supplementary material for: An evidence-based tailored eHealth patient education tool for patients with knee osteoarthritis: protocol for a randomized controlled trial
Source: BMC Musculoskelet Disord. 2022 Mar 22;23:274. doi: 10.1186/s12891-022-05212-0 (PMC8939096; doi:10.1186/s12891-022-05212-0)
Supplement: Supplementary file 4 — Additional file 4. [file 12891_2022_5212_MOESM4_ESM.doc]

**Patient knowledge questionnaire (PKQ-OA)**

**Translated version (original in Chinese)**

**Patient knowledge questionnaire (PKQ-OA)** (Ticked the corresponding ○; marked with * means multiple choice question)

1. Name：

2. Gender？

| ○Male | ○Female |  |  |  |  |  |  |
| --- | --- | --- | --- | --- | --- | --- | --- |

3. Telephone number:

4. Academic degree？

| ○Junior high school and below |
| --- |
| ○High school |
| ○Higher Vocational or Junior College |
| ○Undergraduate |
| ○Master |
| ○Ph.D. or higher |

5. Are you currently having an occupation?

| ○Yes (Skip to question 8 ) |
| --- |
| ○No (Skip to question 6) |

6. If you do not have a job, what is your situation?

| ○Unemployed (Skip to question 7) |
| --- |
| ○Retired (Skip to question 7) |
| ○Loss the ability to work due to illness or disability (Skip to question 7) |

7. If you do not have a job, is the reason related to your knee problems?

| ○Yes (Skip to question 8) |
| --- |
| ○No (Skip to question 8) |

8. How tall are you? (Fill in "centimeter (cm)" as the unit)

9. What is your weight? (Fill in "kilogram or kilogram (Kg)" as the unit)

10. What was your total household income last year? (Fill in "yuan" as the unit)

11. * What treatments have you received so far for knee osteoarthritis?

| □ Physiotherapy (Tuina, joint mobilization, manipulation, thermotherapy and physical agent therapy, etc.) |
| --- |
| □ Traditional Chinese medicine treatment (such as acupuncture, moxibustion, small needle knife, etc.) |
| □ Assistive devices |
| □ Corticosteroid injection |
| □ Arthroscopy |
| □ Non-steroidal anti-inflammatory drugs (such as ibuprofen, aspirin, naproxen) |
| □ Others |

12. Can you choose two true statements from the following list?

□ OA may be triggered by a bacteria or virus

□ OA is caused by cold damp weather

□ OA can be caused by an allergy to some foods

□ OA affects older people

□ OA affects joints that are already damaged in some way

□ Don’t know

13. Can you choose two true statements from the following list?

□ OA only affects the bones of the body

□ OA can cause wear of cartilage

□ OA is curable

□ OA is a chronic disease

□ OA is caused by too much acid in the joints

□ Don’t know

14. Can you choose three symptoms of OA?

□ Loss of movement in a joint

□ Joint stiffness

□ High blood pressure

□ Increase in appetite

□ Breathlessness

□ Aches and pains

□ Don’t know

15. Can you choose two methods used to diagnose OA?

□ Blood tests

□ X-rays

□ Measuring your joint

□ Examining your joints

□ Taking your blood pressure

□ Don’t know

16. Can you choose two correct statements about the predisposing factors of osteoarthritis?

□ Diet

□ Overweight or obesity

□ High blood pressure

□ Depression

□ Overuse

□ Don't know

17. As far as you know, for knee osteoarthritis patients, is therapeutic exercises (resistance training, walking, Tai Chi, etc.) recommended by the Osteoarthritis Research Society International and the Chinese Orthopaedic Association as the primary treatment?

□ Yes

□ No

□ Don't know or cannot draw conclusion

□ Not sure

18. As far as you know, for knee osteoarthritis patients, is losing weight recommended by the Osteoarthritis Research Society International and the Chinese Orthopaedic Association as the primary treatment?

□ Yes

□ No

□ Don't know or cannot draw conclusion

□ Not sure

19. As far as you know, for knee osteoarthritis patients, is strength training for lower limbs recommended by the Osteoarthritis Research Society International and the Chinese Orthopaedic Association as the primary treatment?

□ Yes

□ No

□ Don't know or cannot draw conclusion

□ Not sure

20. As far as you know, for knee osteoarthritis patients, is water aerobic exercise (swimming, etc.) recommended by the Osteoarthritis Research Society International and the Chinese Orthopaedic Association as the primary treatment?

□ Yes

□ No

□ Don't know or cannot draw conclusion

□ Not sure

21. As far as you know, for knee osteoarthritis patients, is using assistive devices (knee pads, insoles, etc.) recommended by the Osteoarthritis Research Society International and the Chinese Orthopaedic Association as the primary treatment?

□ Yes

□ No

□ Don't know or cannot draw conclusion

□ Not sure

22. As far as you know, for knee osteoarthritis patients, is using crutches or walkers recommended by the Osteoarthritis Research Society International and the Chinese Orthopaedic Association as the primary treatment?

□ Yes

□ No

□ Don't know or cannot draw conclusion

□ Not sure

23. As far as you know, for knee osteoarthritis patients, is taking oral and using topical analgesics (nonsteroidal anti-inflammatory medicine, duloxetine, etc.) recommended by the Osteoarthritis Research Society International and the Chinese Orthopaedic Association as the primary treatment?

□ Yes

□ No

□ Don't know or cannot draw conclusion

□ Not sure

24. As far as you know, for knee osteoarthritis patients, is intra-articular injection of glucocorticoids recommended by the Osteoarthritis Research Society International and the Chinese Orthopaedic Association as the primary treatment?

□ Yes

□ No

□ Don't know or cannot draw conclusion

□ Not sure

25. As far as you know, for knee osteoarthritis patients, is self-management and patient education recommended by the Osteoarthritis Research Society International and the Chinese Orthopaedic Association as the primary treatment?

□ Yes

□ No

□ Don't know or cannot draw conclusion

□ Not sure

26. As far as you know, for knee osteoarthritis patients, is thermotherapy recommended by the Osteoarthritis Research Society International and the Chinese Orthopaedic Association as the primary treatment?

□ Yes

□ No

□ Don't know or cannot draw conclusion

□ Not sure

27. As far as you know, for knee osteoarthritis patients who combined with other underlying diseases (diabetes, hypertension, renal impairment, gastrointestinal diseases, anxiety or obesity, etc.), which one of the following treatments is not recommended?

□ Using assistive devices (knee pads, insoles, etc.)

□ Using crutches or walkers

□ Taking oral analgesics

□ Using topical analgesics

□ Don't know

28. As far as you know, for polyarticular osteoarthritis patients who combined with other underlying diseases (diabetes, hypertension, renal impairment, gastrointestinal diseases, anxiety or obesity, etc.), which one of the following treatments is not recommended?

□ Using assistive devices (knee pads, insoles, etc.)

□ Using crutches or walkers

□ Using topical analgesics

□ Intra-articular injection of glucocorticoids

□ Don't know

29. Can you choose two correct answers about exercise and OA?

□ It is not necessary to exercise if you are normally active

□ Exercise will cure OA

□ Exercise weakens damaged joints

□ Exercise strengthens muscles and ligaments

□ Exercise should be taken regularly each day

□ Don’t know

30. Can you choose the two forms of exercise suitable for someone with osteoarthritis?

□ Muscle strengthening exercises

□ Jogging

□ Swimming

□ Weight lifting

□ Housework

□ Don’t know

31. Can you choose one method of reducing the chances of your osteoarthritis getting worse?

□ Exercise vigorously

□ Take your drug therapy regularly

□ Keep your weight down to the ideal for your height and age

□ Rest for most of the time

□ Don’t know

**Patient satisfaction for knee osteoarthritis education (given to eHealth intervention group only)**

**Translated version (original in Chinese)**

1. To what degree of the eHealth patient education tool did you used? [Single choice]

□ None

□ Some

□ Most

□ All

2. When you see a doctor for other diseases, do you think the similar materials and tools (such as posters or brochures, etc.) should be provided during the process? [Single choice]

□ Yes

□ No

□ Not sure

3. Do you think the eHealth patient education tool useful to help receive the treatment options your provider talks about? [Single choice]

□ Yes

□ No

□ Not sure

4. If applicable, do you believe you were able to remember more about treatments for knee osteoarthritis because you used eHealth patient education tool? [Single choice]

□ Yes

□ No

□ Not sure

5. If applicable, did the eHealth patient education tool help you trust the advice your doctor provided? [Single choice]

□ Yes

□ No

□ Not sure

6. If applicable, are you more likely to follow your doctor’s advice after using the eHealth patient education tool? [Single choice]

□ Yes

□ No

□ Not sure

7. In the range of 0 to 10 points, 0 means very dissatisfied and 10 means very satisfied. How satisfied are you with the experience of using the eHealth patient education tool?

|  | 1 | 2 | 3 | 4 | 5 | 6 | 7 | 8 | 9 | 10 |  |
| --- | --- | --- | --- | --- | --- | --- | --- | --- | --- | --- | --- |
| Very dissatisfied | ○ | ○ | ○ | ○ | ○ | ○ | ○ | ○ | ○ | ○ | Very satisfied |

8. How would you comment on this eHealth patient education tool?

**System Usability Scale (SUS) (given to eHealth intervention group only)**

**Translated version (original in Chinese)**

**System Usability Scale (SUS)**

Please select the answer that best expresses how you feel about each statement after using the eHealth tool (In the range of 1 to 5 points, 0 means strongly disagree and 10 strongly agree)

|  | 1 | 2 | 3 | 4 | 5 |
| --- | --- | --- | --- | --- | --- |
| 1. I found it simple to use the eHealth tool. | ○ | ○ | ○ | ○ | ○ |
| 1. I thought it was easy to learn to use this eHealth tool. | ○ | ○ | ○ | ○ | ○ |
| 1. I think that I would need the support of a technical person to be able to use this eHealth tool. | ○ | ○ | ○ | ○ | ○ |
| 1. I think I would like to use this eHealth tool frequently. | ○ | ○ | ○ | ○ | ○ |
| 1. The eHealth tool gives error messages that clearly tell me how to operate correctly. | ○ | ○ | ○ | ○ | ○ |
| 1. I found the various functions and information in this tool were well integrated as I expected. | ○ | ○ | ○ | ○ | ○ |
| 1. I think it is easy to find the information I needed. | ○ | ○ | ○ | ○ | ○ |
| 1. I found the information provided with the eHealth tool is effective in helping me get well knowledge of patient education. | ○ | ○ | ○ | ○ | ○ |
| 1. I found the organization of information on the eHealth tool screens is clear. | ○ | ○ | ○ | ○ | ○ |
| 1. I found the interface of this eHealth tool is pleasant. | ○ | ○ | ○ | ○ | ○ |
| 1. I felt very confident using the eHealth tool. | ○ | ○ | ○ | ○ | ○ |
| 1. I needed to learn a lot of things before I could get going with this tool. | ○ | ○ | ○ | ○ | ○ |
| 1. Overall, I am satisfied with this eHealth tool. | ○ | ○ | ○ | ○ | ○ |

**Open questions**

1. How likely are you to recommend this eHealth tool to others? (please circle your answer)

Not at all likely 0 1 2 3 4 5 6 7 8 9 10 Extremely likely

2. Please briefly describe how useful this tool is during your consultation with a healthcare provider?

3. Any advice on how we can improve your experience with the use of the eHealth tool?
